# A Graph-Neural-Network-Based Social Network Recommendation Algorithm Using High-Order Neighbor Information

**DOI:** 10.3390/s22197122

**Published:** 2022-09-20

**Authors:** Yonghong Yu, Weiwen Qian, Li Zhang, Rong Gao

**Affiliations:** 1 College of Tongda, Nanjing University of Posts and Telecommunication, Yangzhou 225127, China; 2 Department of Computer Science, Royal Holloway, University of London, Egham TW20 0EX, UK; 3 School of Computer Science, Hubei University of Technology, Wuhan 430068, China

**Keywords:** recommendation algorithm, graph neural network, high-order neighbors, social network

## Abstract

Social-network-based recommendation algorithms leverage rich social network information to alleviate the problem of data sparsity and boost the recommendation performance. However, traditional social-network-based recommendation algorithms ignore high-order collaborative signals or only consider the first-order collaborative signal when learning users’ and items’ latent representations, resulting in suboptimal recommendation performance. In this paper, we propose a graph neural network (GNN)-based social recommendation model that utilizes the GNN framework to capture high-order collaborative signals in the process of learning the latent representations of users and items. Specifically, we formulate the representations of entities, i.e., users and items, by stacking multiple embedding propagation layers to recursively aggregate multi-hop neighborhood information on both the user–item interaction graph and the social network graph. Hence, the collaborative signals hidden in both the user–item interaction graph and the social network graph are explicitly injected into the final representations of entities. Moreover, we ease the training process of the proposed GNN-based social recommendation model and alleviate overfitting by adopting a lightweight GNN framework that only retains the neighborhood aggregation component and abandons the feature transformation and nonlinear activation components. The experimental results on two real-world datasets show that our proposed GNN-based social recommendation method outperforms the state-of-the-art recommendation algorithms.

## 1. Introduction

With the emergence and prosperity of online service platforms, the accumulated data provide users with rich information resources. However, the massive amounts of data also hinder users from finding the content in which they are interested, leading to the problem of information overload. A recommendation system (RS) [1] is an effective tool for alleviating information overload. An RS analyzes the data on a user’s historical behavior, mines the user’s potential preferences, and provides them with personalized services. RSs have been widely deployed in industrial applications, since they can not only improve user experiences, but also increase revenue for the providers of online service platforms.

The collaborative filtering (CF) algorithm [2,3] is the most widely used technology for building recommendation systems, and it has achieved great success in various online service platforms. CF basically assumes that users with similar behaviors will have similar preferences for items. However, CF seriously suffers from the issue of data sparsity. In order to deal with this issue and improve the performance of recommendation, researchers have proposed some social-network-based recommendation algorithms (SRSs) [4,5,6,7,8] by integrating social network information into traditional recommendation models. In general, an SRS utilizes social network information to make a user and his/her friends have similar preference representations. These traditional SRS models, such as SoRec [4], RSTE [5], SocialMF [6], TrustMF [7], and TrustSVD [8], basically employ regularization terms to guide the process of user preference representations. Specifically, they usually provide a constraint so that the difference in preference representations between a user and his/her friends is relatively small. However, it is not intuitive for these social-regularization-based models to utilize the structural information of social networks. In addition, collaborative signals between friends are not effectively captured in the process of learning user preferences. As reported by [9,10,11], the integration of collaborative signals into recommendation models is able to improve the performance of the recommendations.

Recently, graph neural networks (GNNs) [12,13,14] have been widely adopted in many fields, such as natural language processing (NLP) and computer vision (CV), due to their convincing performance and high interpretability. In the field of recommendation systems, some researchers have also utilized GNNs to improve the performance of social-network-based recommendation models [11,15]. Generally, these GNN-based social network recommendation models adopt the core operations of GNNs, i.e., feature transformation and nonlinear activation, on the social network to learn users’ representations. However, most existing GNN-based recommendation models only take first-order collaborative signals into consideration, but ignore higher-order collaborative signals. In other words, when aggregating neighbors’ preference representations, they only consider the direct neighbors and neglect the multi-hop neighbors. On the other hand, some GNN-based recommendation models [16] exploit higher-order social relations to improve recommendation performance, but they overlook the higher-order collaborative signals derived from user–item interaction graphs.

In this paper, we propose a GNN-based social recommendation model that utilizes GNNs to capture high-order collaborative signals when learning latent representations of users and items. Specifically, there are two interaction graphs in the social-network-based recommender system, i.e., a user–item interaction graph and a social network graph. For each interaction graph, we represent nodes by iteratively aggregating the representations of multi-hop neighbors. In this way, we simultaneously capture two types of collaborative signals for users, which are embedded in interaction-specific and social-specific latent representations, respectively. For the items, we use the interaction-specific latent representations to capture collaborative signals hidden in the user–item interaction graph. Hence, the aggregated latent representations of nodes encode rich semantics, which can better characterize user preferences and item characteristics. Moreover, our proposed GNN-based social recommendation model is a lightweight model that abandons the feature transformation and nonlinear activation components. The lightweight scheme eases the training process of the proposed recommendation model and alleviates the problem of overfitting.

Our major contributions can be summarized as follows:

- We propose a GNN-based social recommendation model that utilizes GNNs to capture high-order collaborative signals hidden in the user–item interaction graph and user–user social graph.
- In order to alleviate overfitting and ease the computational costs of model training, we simplify the GNNs by abandoning the feature transformation and nonlinear activation components when integrating GNNs into the social network recommendation model.
- We conducted extensive experiments to evaluate our proposed GNN-based social network recommendation model on real-life datasets. The experimental results show that our proposed method is superior to traditional recommendation methods.

The remainder of this paper is summarized as follows. We first briefly review the typical related work in Section 2. Section 3 illustrates the definition of the problem of social-network-based recommendation systems. In Section 4, we describe the details of our proposed GNN-based social network recommendation method. In Section 5, we describe the extensive experiments that were performed to evaluate the effectiveness of our proposed model. Finally, we conclude our work and present future directions in Section 6.

## 2. Related Work

This section briefly reviews the state-of-the-art related works, including those on social network-based recommendation algorithms and GNN-based recommendation algorithms.

### 2.1. Social-Network-Based Recommendation Methods

With the emergence of social networks, researchers have proposed a variety of recommendation algorithms based on them that utilize the structural information embedded in the social networks to alleviate the cold-start problem. In fact, social-network-based recommendation algorithms are inspired by the social influence theory [17,18]. Social-network-based recommendation methods usually extend the classic matrix factorization model by integrating social network information into a user–item rating matrix. For example, Ma et al. [4] proposed SoRec, which fuses the user–item rating matrix with the users’ social network using PMF. In order to connect two different data sources, SoRec shares the user’s latent feature matrix in both the social network structure and the user–item rating matrix. Based on the intuition that one user’s final decision should be affected by both the user’s own preferences and the user’s trusted friends’ recommendations, Ma et al. [5] proposed a probabilistic factor analysis framework called RSTE. Specifically, RSTE naturally fuses the user’s tastes and their trusted friends’ endorsements by using an ensemble parameter. Since the transitivity of trust and trust propagation are not well captured by RSTE, Jamali et al. [6] proposed a model-based approach for recommendation in social networks, namely, SocialMF. SocialMF incorporates the mechanism of trust propagation into the matrix factorization model, where the feature vector of each user is dependent on the feature vectors of their direct neighbors in the social network. Motivated by the heuristic that individuals will affect each other during the process of reviewing, Yang et al. [7] proposed a social CF method named TrustMF. TrustMF maps users into two low-dimensional spaces, i.e., truster space and trustee space, by factorizing the trust network according to the directional properties of trust. The two feature vectors learned from the truster and trustee spaces for each user explicitly describe how users affect or follow the opinions of others, respectively. On top of SVD++ [19], which considers explicit and implicit influences of ratings when generating predictions, Guo et al. [8] proposed a trust-based recommendation model, TrustSVD. TrustSVD extends the SVD++ model by further incorporating both the explicit and implicit influences of trusted users on the prediction of items for an active user.

The results of the representative methods presented above and other social-network-based recommendation methods [20,21] show that social network information is beneficial for item recommendation. One crucial reason is that rich social network information is able to alleviate the data sparsity and cold-start problems, which seriously degrade the performance of recommender systems.

### 2.2. GNN-Based Recommendation Methods

Due to GNNs’ convincing performance and high interpretability, they have been widely adopted in some machine learning tasks that are related to graph analysis, such as social network prediction [12], traffic prediction [22], and graph representation [23]. Recently, some researchers also adopted GNNs in order to extend traditional recommendation models, and they proposed several GNN-based recommendation methods. Representative GNN-based recommendation methods include SR-GNN [24], NGCF [10], LightGCN [9], GraphRec [15], and DiffNet [11]. In [24], Wu et al. proposed a session-based recommendation model using GNNs, which was called SR-GNN. SR-GNN separates session sequences into graph-structured data and uses GNNs to capture complex item transitions. To explicitly encode collaborative signals in the process of learning the embeddings of nodes, Wang et al. [10] proposed a neural graph collaborative filtering model called NGCF. Specifically, NGCF captures collaborative signals through multi-layer embedding propagation operations, resulting in the expressive modeling of high-order connectivity in the user–item graph. In order to make GNNs more appropriate for recommendation, He et al. [9] further proposed a lightweight GNN-based recommendation model named LightGCN, which only retains the most essential component of GCNs, i.e., neighborhood aggregation, for collaborative filtering. Note that NGCF and LightGCN only utilize the user–item rating matrix to learn their model parameters. For users or items with few or even no ratings, they also encounter the cold-start problem. Hence, inspired by traditional social-network-based recommendation models, some researchers utilized social network information and took advantage of GNNs to boost the performance in recommendation. For example, Fan et al. [15] proposed a GNN framework for social recommendation, i.e., GraphRec, which coherently models graph data in social recommendations. In addition, GraphRec differentiates the tie strengths of social relations via the attention mechanism. To stimulate the recursive social influence propagation process for better modeling of user and item embedding, Wu et al. [11] proposed an influence diffusion neural network for a social recommendation model, DiffNet. DiffNet leverages the graph convolution operation for the recursive social diffusion in the social networks. To model the indirect influence from the high-order neighbors in social networks, Liu et al. [16] proposed a novel social recommendation framework named HOSR, which explicitly encodes high-order social relations into the learning of user embeddings through multi-step message propagation. Unlike HOSR, which refines user embeddings by capturing high-order collaborative signals encoded in the social network, our proposed social-network-based recommendation model simultaneously captures both types of collaborative signals, which are hidden in the user–item interaction graph and social network graph, respectively.

GraphRec is one of most relevant works to our proposed method. The main differences between GraphRec and our proposed model lie in the following aspects: (1) When aggregating neighbors’ embeddings, GraphRec only considers the representations of directly connected neighbors in both the user–item interaction graph and social network graph, while our proposed social recommendation model refines the nodes’ representations by recursively aggregating multi-hop neighbors’ representations. In this way, our proposed model is able to explicitly inject high-order collaborative filtering signals encoded in the two graphs during the process of learning users’ preferences and items’ characteristics. (2) GraphRec completely adopts three core components of GNNs, i.e., feature transformation, nonlinear activation, and neighborhood aggregation, to refine the nodes’ representations, but our proposed model only retains the neighborhood aggregation operation, since it is reported that the other two components are not beneficial for recommendation [9,25]. Hence, our proposed GNN-based social recommendation model is a lightweight model. Another closely related study is that on SocialLGN [26], which extends the LightGCN to make it more adaptable to the social recommendation problem. Although both SocialLGN and our proposed model extend LightGCN to deal with the social recommendation problem, the main differences between them are twofold: (1) SocialLGN mainly focuses on the Top-N recommendation task, while our proposed method focuses on the rating prediction task. In other words, SocialLGN is designed for users’ implicit feedback, and our proposed method directly learns users’ latent preferences from users’ explicit feedback. (2) In the prediction layer, unlike SocialLGN, which leverages the inner product between the weighted user representation and the item representation to calculate the ranking scores, our proposed method feeds the concatenation of the final representations of users and items into a multi-layer perceptron to predict the ratings. Adopting the multi-layer perceptron enables our proposed model to capture the complex interactive behaviors between each dimension of the final representations of the users and items.

## 3. Problem Definition

In this paper, we focus on social-network-based recommendation with explicit rating information. Hence, we describe the definition of social-network-based recommendation in the following. Generally, social-network-based recommendation systems contain two different types of data sources: the user–item rating matrix and the social network information. The user–item rating matrix $R\in R^{\left|U|\times |I\right|}$ usually consists of two entity sets: the user sets $U=\{u_{1},u_{2},\ldots ,u_{\left|U\right|}\}$ and the item sets $I=\{i_{1},i_{2},\ldots ,i_{\left|I\right|}\}$. Each entry $r_{ui}$ of *R* is the rating on item *i* assigned by user *u*. The higher the rating $r_{ui}$ is, the more satisfied the user *u* is with the item *i*.

Meanwhile, the social network information is usually expressed as a directed relation graph G=(U,E), where *E* is the set of trust relationships between users. The trust relationships among all users form a trust relationship matrix $T\in R^{\left|U|\times |U\right|}$. The element $t_{u,v}\in \left[0,1\right]$ denotes the trust strength between the user *u* and the user *v*. $t_{u,v}$ = 0 means that there is no trust relationship between the user *u* and the user *v*. In real social networks, the trust relationships between users tend to be one-way. Hence, the trust relationship matrix *T* is often asymmetric. In social-network-based recommendation, social relationship information is often a beneficial complement to the user–item ratings and helps recommendation models to more accurately infer user preferences and item characteristics.

The goal of a social-network-based recommendation system is firstly to predict the ratings on candidate items for the active user by utilizing both the user–item rating matrix and the social relationship matrix. Then, the recommendation model provides a ranked list of items for the active user according to the predicted ratings.

## 4. The Proposed Method

In this section, we first describe the overall framework of our proposed model, followed by a presentation of each component in detail. Finally, we introduce the process of learning the model parameters.

### 4.1. The Framework of Our Proposed Method

**Figure 1:**
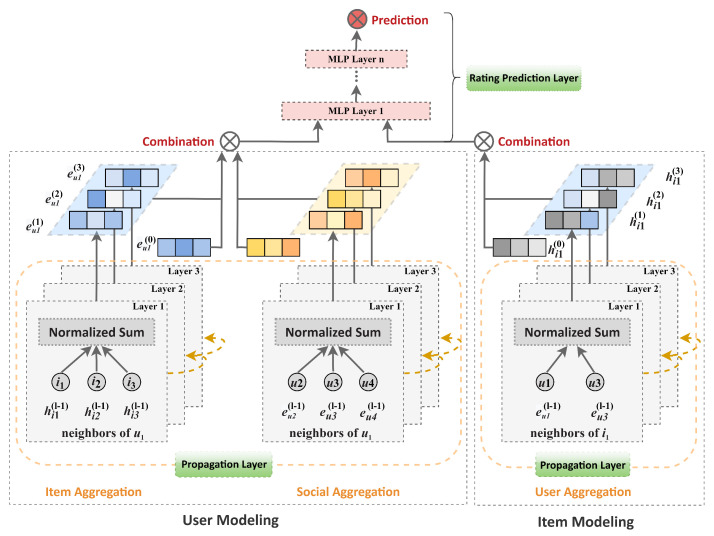
The framework of the proposed recommendation model.

The overall framework of our proposed model is illustrated in Figure 1. The model contains three main parts: the embedding layer, propagation layers, and rating prediction layer. In the embedding layer, we associate each user and item with an embedding according their IDs, initializing with a Gaussian distribution. In the propagation layers, our model recursively performs an aggregation operation, which forms a node’s representation by fusing the neighbors’ feature representations in both the user–item interaction graph and social network graph. In the rating prediction layer, we feed an active user’s final embedding and a target item’s final embedding into a multi-layer perceptron and use the multi-layer perceptron to predict the rating assigned by the active user to the target item. We introduce each component in detail in the following subsections.

### 4.2. Embedding Layer

In the embedding layer, we map users and items into a low-dimensional latent space according to their IDs. Suppose that *u* and *i* indicate the indexes of user *u* and item *i*, respectively. We obtain the basic embeddings of user *u* and item *i* with an embedding table lookup operation. Formally,

(1) $$\begin{array}{c} e_{u}=U.onehot\left(u\right),\;h_{i}=I.onehot\left(i\right), \end{array}$$

where onehot(.) denotes the one-hot encoding operation. $U\in R^{K\times |U|}$ and $I\in R^{K\times |I|}$ represent the user embedding matrix and the item embedding matrix, respectively, where *K* is the dimension of latent embedding.

### 4.3. Embedding Propagation Layers

The propagation layers mainly include two parts: user modeling and item modeling. The aim of these two components is to cooperatively learn users’ and items’ embeddings by capturing high-order collaborative signals that are hidden in the user–item interaction graph and the social network. We present the details of user modeling and item modeling in Section 4.3.1 and Section 4.3.2, respectively.

#### 4.3.1. User Modeling

The goal of user modeling is to learn the user’s embedding over the user–item interaction graph and the user-user social graph. The main challenge of user modeling is that of capturing high-order collaborative signals when learning the users’ embeddings. To address this challenge, as illustrated on the left side of Figure 1, we exploit a lightweight GNN framework to explicitly capture the collaborative signals encoded in these two graphs by recursively propagating neighbors’ representations. Specifically, we perform two types of neighborhood aggregations, i.e., item aggregation and social aggregation, on the user–item interaction graph and social network graph, respectively. The item aggregation mainly captures collaborative signals between users and items, which are encoded in the user–item interaction graph. On the other hand, the social aggregation captures collaborative signals among users, which are encoded in the social network graph.

Formally, with *l* embedding propagating layers, the abstract representation of user *u* is formulated as:

(2) $$e_{u}^{\left(l\right)}=Agg_{u}\left(\left\{h_{i}^{\left(l-1\right)},\forall i\in N_{u}^{I}\right\},\left\{e_{f}^{\left(l-1\right)},\forall f\in N_{u}^{S}\right\}\right)$$

where $e_{u}^{\left(l-1\right)}$ and $h_{i}^{\left(l-1\right)}$ denote the embeddings of user *u* and item *i* that are obtained at the (l−1)-th embedding propagation layer. $Agg_{u}\left(*\right)$ is the user aggregation function, which aggregates two types of representations, i.e., the representations of items that interact with user *u* and the representations of users that are trusted by user *u*. $N_{u}^{I}$ denotes the set of items that interact with user *u*, and $N_{u}^{S}$ is the set of users trusted by user *u*. In our proposed recommendation model, we adopt the weight sum as the user aggregation function:

(3) $$e_{u}^{\left(l\right)}=\sum_{i\in N_{u}^{I}}\frac{1}{\sqrt{\left|N_{u}^{I}\right|}\sqrt{\left|N_{i}^{I}\right|}}h_{i}^{\left(l-1\right)}+\sum_{f\in N_{u}^{S}}\frac{1}{\sqrt{\left|N_{u}^{S}\right|}\sqrt{\left|N_{f}^{S}\right|}}e_{f}^{\left(l-1\right)}$$

where $N_{i}^{I}$ denotes the set of users who interact with item *i*. $\frac{1}{\sqrt{\left|N_{u}^{I}\right|}\sqrt{\left|N_{i}^{I}\right|}}$ and $\frac{1}{\sqrt{\left|N_{u}^{S}\right|}\sqrt{\left|N_{f}^{S}\right|}}$ are symmetric square root normalization terms, which are used to avoid the amplification of the embedding scale caused by the graph convolution operation.

As shown in Equation (3), the model that we propose is a lightweight GNN framework that abandons the feature transformation and nonlinear activation operations. The scheme of the lightweight design is inspired by LightGCN [9] and SGC [25]. The reason for simplifying the classic GNN framework is that the feature transformation and nonlinear activation operations increase the difficulty of training GNN-based recommendation models, resulting in poor generalizability. In fact, the feature transformation and nonlinear activation operations have limited contributions to recommendation performance, as evidenced in [9]. Moreover, without the feature transformation and nonlinear activation operations, the user modeling described by Equation (3) is much easier to implement and train.

#### 4.3.2. Item Modeling

Since items are only involved in the user–item interaction graph, item modeling aggregates the representations of neighbors who interact with the item when learning item representations. Similar to user modeling, we utilize lightweight GNNs to capture the collaborative signal by recursively propagating representations of neighbors over the user–item interaction graph.

As illustrated on the right side of Figure 1, the item modeling operation can be abstractly expressed as:

(4) $$h_{i}^{\left(l\right)}=Agg_{i}\left(\left\{e_{u}^{\left(l-1\right)},\forall u\in N_{i}^{I}\right\}\right)$$

where $Agg_{i}\left(*\right)$ denotes the item aggregation function, which aggregates the neighbor nodes of item *i*.

We adopt the weight sum with symmetric square root normalization to compute the embedding of item *i* at the *l*-th propagation layer:

(5) $$h_{i}^{\left(l\right)}=\sum_{u\in N_{i}^{I}}\frac{1}{\sqrt{\left|N_{i}^{I}\right|}\sqrt{\left|N_{u}^{I}\right|}}e_{u}^{\left(l-1\right)}$$

According to Equations (3) and (5), the high-order collaborative signals are injected into the representations of users and items, respectively, by performing multiple neighborhood aggregation operations in a recursive manner. For example, $h_{i}^{\left(1\right)}$ captures the first-order connectivity information over the user–item interaction graph. On top of the neighbor’s embedding $h_{i}^{\left(1\right)}$, $h_{i}^{\left(2\right)}$ encodes the two-order connectivity information by aggregating the representations of neighbors that are learned in the first propagation layer. In this way, $h_{i}^{\left(l\right)}$ considers the *l*-hop neighborhood information, since $h_{i}^{\left(l\right)}$ is the weight sum of the neighbors’ embeddings that are learned in the (l−1)-th propagation layer. Similarly to $h_{i}^{\left(l\right)}$, $e_{u}^{\left(l\right)}$ simultaneously captures *l*-hop collaborative signals over both the user–item interaction graph and social network graph.

### 4.4. Rating Prediction Layer

With *l* embedding propagation layers, we obtain the set of user embeddings $\left(e_{u}^{\left(0\right)},e_{u}^{\left(1\right)},\ldots ,e_{u}^{\left(l\right)}\right)$ and the set of item embeddings $\left(h_{i}^{\left(1\right)},\ldots ,h_{i}^{\left(l\right)}\right)$ for user *u* and item *i*, respectively. Each element $e_{u}^{\left(.\right)}$ or $h_{i}^{\left(.\right)}$ encodes a different order of connectivity information and describes different aspects of user preferences and item characteristics. For each user or item, in order to capture both lower-order and higher-order collaborative signals among users and items, we combine all corresponding elements to obtain the final representation as follows:

(6) $$e_{u}^{*}=\sum_{l=0}^{L}a_{l}e_{u}^{\left(l\right)},h_{i}^{*}=\sum_{l=0}^{L}a_{l}h_{i}^{\left(l\right)}$$

where *L* denotes the number of embedding propagation layers. $a_{l}$ denotes the weight of the embedding of users or items in the *l*-th layer. In order to simplify our model, we empirically set $a_{l}$ to $\frac{1}{\left(L+1\right)}$. In fact, we also attempt to automatically learn the weight parameter by utilizing the attention network. However, as compared with the simple weight assignment scheme, the recommendation performance achieved by integrating the attention network does not show a significant margin. By using Equation (6) to compute the final embeddings of users or items, the final representations are endowed with rich semantics, increasing the expressive power of users’ and items’ embeddings.

After we obtain the final embeddings $e_{u}^{*}$ and $h_{i}^{*}$, we concatenate $e_{u}^{*}$ and $h_{i}^{*}$ as $\left[e_{u}^{*}⊕h_{i}^{*}\right]$ and feed $\left[e_{u}^{*}⊕h_{i}^{*}\right]$ into a multi-layer perceptron (MLP) with a tower structure to predict the rating of item *i* given by user *u*:

(7) $${\hat{y}}_{ui}=MLP\left(\left[e_{u}^{*}⊕h_{i}^{*}\right]\right)$$

where ${\hat{y}}_{ui}$ is the predicted rating of user *u* on item *i*, and ⊕ denotes the concatenation operation. MLP(*) is a multi-layer perceptron with a tower structure. Differing from traditional matrix-factorization-based recommendation models that use the dot product between the user embedding and item embedding to predict ${\hat{y}}_{ui}$, an MLP is adopted to compute ${\hat{y}}_{ui}$, since it is able to approximate any interaction function between a user and an item. Moreover, taking the MLP as the interaction function allows the complex interactive behaviors between each dimension of $e_{u}^{*}$ and $h_{i}^{*}$ to be captured.

### 4.5. Model Training

In general, there are two types of tasks in the field of recommendation systems, i.e., rating prediction and ranking. Since our work focuses on the task of rating prediction, we take the sum of squared errors with quadratic regularization terms as our objective functions, formally, as follows.

(8) $$L=\frac{1}{2|\Omega |}\sum_{u,i\in \Omega }({\hat{y}}_{ui}-y_{ui}{)}^{2}+\lambda {∥\Theta ∥}_{F}^{2}$$

where Ω is the set of (u,i) pairs for the observed ratings, and ${∥.∥}_{F}^{2}$ is the Frobenius norm. $y_{ui}$ indicates the actual rating of user *u* on item *i*, and ${\hat{y}}_{ui}$ indicates the predicted rating of user *u* on item *i*. λ is the regularization coefficient, which controls the influence of the regularization term. In our proposed recommendation model, the model parameter is $\Theta =\{U,I,W^{\left(m\right)},b^{\left(m\right)},m=1,2,\ldots ,M\}$, where $W^{\left(m\right)}$ and $b^{\left(m\right)}$ indicate the weight matrix and bias vector for the *m*-th hidden layer within the MLP, respectively, with *m* denoting the number of hidden layers for the MLP. In order to learn the model parameters, we use the Adam optimizer to optimize the parameters. In addition, we initialize all trainable parameters using a Gaussian distribution with a mean of 0 and a standard deviation of 0.01.

## 5. Empirical Analysis

In this section, we describe several experiments that we conducted on real datasets to evaluate our proposed GNN-based social recommendation model. We denote our proposed lightweight GNN-based social recommendation model as Light_NGSR.

### 5.1. Dataset

We chose the Ciao (http://www.ciao.co.uk) and Epinions ( http://www.epinions.com) datasets to evaluate the effectiveness of our proposed recommendation algorithm, since both Ciao and Epinions contain rating and social network information, and they are widely used to verify the performance of social-network-based recommendation algorithms. In both Ciao and Epinions, users utilize ratings and reviews to express their opinions on items, where each rating is an integer that ranges from 1 to 5. Meanwhile, each user maintains a list of friends that they trust. Note that the values of trust between users are binary, and the trust relationships are directed. The statistics of the Ciao and Epinions datasets are shown in Table 1.

### 5.2. Metrics

In this research, the root mean squared error (RMSE ) and mean absolute value error (MAE ) were used to measure the recommendation quality of our proposed model. Formally, the RMSE and MAE are defined as follows:

(9) $$RMSE=\sqrt{\frac{\sum_{\left(u,i\right)\in R_{test}}{\left|y_{ui}-{\hat{y}}_{ui}\right|}^{2}}{\left|R_{test}\right|}}$$

(10) $$MAE=\frac{\sum_{\left(u,i\right)\in R_{test}}\left|y_{ui}-{\hat{y}}_{ui}\right|}{\left|R_{test}\right|}$$

where $R_{test}$ denotes the set of user–item pairs in the test dataset. As shown in Equations (9) and (10), the smaller the RMSE and MAE are, the better the performance of the recommendation algorithm is.

### 5.3. Baselines and Parameter Settings

In order to evaluate the effectiveness of our proposed recommendation algorithm, we chose the following recommendation algorithms as baselines:

(1) PMF: PMF [27] can be regarded as a probabilistic extension of the SVD model. Note that PMF only utilizes a user–item rating matrix to learn the latent user feature vectors and latent item feature vectors.

(2) SoRec: SoRec [4] simultaneously factorizes the user–item rating matrix and the trust relationship matrix by sharing the user latent feature matrix between the trust relationship matrix and the user–item rating matrix. Hence, SoRec is a social-network-based recommendation model.

(3) RSTE: RSTE [5] is also a social-network-based recommendation algorithm. RSTE formulates users’ final decisions by utilizing an ensemble parameter to fuse a user’s own preferences and their friends’ preferences.

(4) SocialMF: SocialMF [6] incorporates the mechanism of trust propagation into the matrix factorization model, where each user is influenced by their direct neighbors in the social network.

(5) TrustMF: TrustMF [7] maps users into truster space and trustee space by factorizing the trust network according to the directional properties of trust. The two feature vectors of each user that are learned from the above two spaces explicitly model how users affect or follow the opinions of others, respectively.

(6) GraphRec: GraphRec [15] is a state-of-the-art GNN framework for social recommendation. GraphRec coherently models graph data and differentiates the tie strengths of social relations via an attention mechanism.

For a fair comparison, we tuned the parameters of each method according to the respective references or based on our experiments. The main parameter settings of all algorithms used for the comparison are listed in Table 2. With these parameter settings, each method achieved its best recommendation quality. In addition to the parameter settings listed in Table 2, we fine-tuned the learning rate η at {0.0001,0.0005,0.001,0.005,0.01,0.05} for all comparison methods. In addition, we empirically set the dimension of latent embedding to 64, i.e., K=64. Moreover, for our proposed recommendation algorithm, we set the number of embedding propagation layers to L=3 and the number of hidden layers of the MLP to M=3.

For both datasets, each time, we conducted a five-fold cross-validation, which randomly selected 80% of the data from the datasets as the training set, with the remaining 20% being used as the test set. Finally, we report the average results on the test set for each dataset.

### 5.4. Performance Comparison

The experimental results of all algorithms used for comparison on the two datasets are presented in Table 3 and Table 4.

From Table 3 and Table 4, we have the following observations: (1) Of all of the compared methods, PMF achieved the worst recommendation performance. This is because PMF learns latent user feature vectors and latent item feature vectors by factorizing the user–item rating matrix, but the user–item rating information is extremely sparse. Hence, PMF was not able to learn more accurate latent user feature vectors and latent item feature vectors, since the training data were not sufficient. This observation is consistent with the results reported by [4,5,6,7,15]. (2) The traditional social-network-based algorithms (e.g., SoRec, RSTE, SocialMF, TrustMF) were superior to PMF on both datasets, demonstrating the advantages of combining social network information and user–item ratings for recommender systems. The main reason is that social networks provide rich information for the basic matrix factorization model and alleviate the problem of data sparsity to some extent. In fact, with the help of social networks, traditional social-network-based algorithms may be able to more compressively infer users’ latent preferences, especially for users who have few or even no ratings. (3) Of the traditional social-network-based recommendation algorithms, SocialMF achieved the best performance. For traditional social-network-based recommendation models, this observation indicates that integrating the mechanism of trust propagation is more effective than sharing the latent user feature matrix between the social network matrix and user–item rating matrix, fusing the user’s and friends’ preferences, or mapping each user into two separate spaces. (4) Except for our proposed social recommendation model, GraphRec outperformed the other baselines, with a significant improvement. This superiority demonstrates the powerful expressiveness of GNNs for social recommendation. We argue that the main reason is that the GraphRec inherits the merits of neighborhood-based recommendation models [8,19,28]. The critical idea of neighborhood-based recommendation models is the integration of the representations of items that are rated by users when predicting the ratings. Intrinsically, GraphRec is also a neighborhood-based recommendation method, as it uses the GNN framework to aggregate direct neighbors’ representations. Unlike traditional neighborhood-based recommendation methods that only integrate the embeddings of rated items to represent a user, GraphRec adopts an additional aggregation of the embeddings of users who interact with an item to formulate the target item. (5) On both datasets, our proposed GNN-based social recommendation method consistently outperformed the other methods used for comparison, which verified the effectiveness of our proposed method. Compared with traditional social-network-based recommendation methods, the model that we proposed achieved the best performance, which further shows the power of GNNs for recommendation systems. In addition, in terms of the RMSE, our proposed method showed performance enhancements of 1.25% and 0.19% on the Ciao and Epinions datasets, respectively, when compared with the best baseline method, i.e., GraphRec. Moreover, in terms of the MAE, the improvements of our proposed method over GraphRec were 4.09% and 1.59% on the Ciao and Epinions datasets, respectively. This observation confirms the assumption that the capture of high-order collaborative signals based on lightweight GNNs is able to improve the recommendation performance. (6) For each method, its recommendation performance was better on Ciao than it was on Epinions. This observation indicates that the performance of the recommendation system can benefit from an increase in the density of the dataset.

### 5.5. The Effect of the Number of Propagation Layers *L*

**Figure 2:**
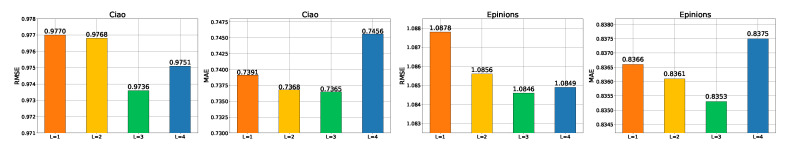
The impact of the number of embedding propagation layers *L*.

Our proposed recommendation model aims to enrich the representations of users and items by using a lightweight GNN to integrate high-order collaborative signals into them. In this section, we describe a group of experiments that we performed to investigate how high-order collaborative signals affect the recommendation performance. We varied the number of embedding propagation layers *L* among the values of [1,2,3,4] and kept the other parameters unchanged. The experimental results are plotted in Figure 2.

As shown in Figure 2, we observed that the recommendation quality of our proposed method was sensitive to the number of embedding propagation layers *L*. In all cases, both the RMSE and MAE first decreased with the increase in the value of *L*, indicating that the recommendation performance was accordingly improved. Then, after the value of *L* passed a certain threshold, the RMSE and MAE moved upward as *L* increased, which meant that the recommendation quality of our proposed recommendation method began to degrade. One possible reason is that the strength of the collaborative signal between a node and its neighbors gradually becomes weak with the increase in the distances between them. Once the collaborative signal has been completely encoded into the representations of users and items, further increasing the value of *L* may not be beneficial for enriching the semantics of representations of users and items, or might even introduce some noise into the representations, thus degrading the recommendation performance. On both datasets, our proposed method yielded the best results in terms of all evaluation metrics when *L* was around 3.

Moreover, as shown in Table 3, Table 4, and Figure 2, even when the number of embedding propagation layers *L* was set to 1, our proposed recommendation method still outperformed GraphRec. Essentially, GraphRec also captured the first-order collaborative signal. However, GraphRec adopts a classic GNN framework, i.e., a heavyweight GNN, to aggregate neighborhood information, while our proposed recommendation model aggregates neighbors’ representations by utilizing a lightweight GNN framework that abandons the feature transformation and nonlinear activation components. This finding is consistent with several recommendation models based on lightweight GNNs [9,29,30], which show that the feature transformation and nonlinear activation components barely contribute to the recommendation quality.

### 5.6. The Effect of the Social Network

**Figure 3:**
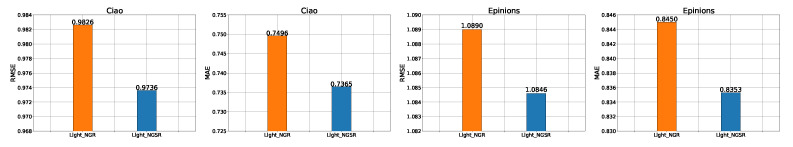
Performance comparison between Light_NGSR and Light_NGR.

Since our proposed model is a social-network-based recommendation model, we conducted an ablation analysis to analyze whether social network information had a contribution to the recommendation quality of our proposed model. We denote the simplified version of Light_NGSR as Light_NGR, which does not utilize the social network information and only makes use of the user–item rating matrix to make recommendations. We evaluated Light_NGSR and Light_NGR on both Ciao and Epinions to investigate the effect of social network information on the recommendation quality. The experimental results are shown in Figure 3.

In Figure 3, we can see that the social network information significantly affected the recommendation quality of our proposed recommendation model. Moreover, on both datasets, Light_NGSR was consistently superior to Light_NGR. In terms of the RMSE, the improvements of Light_NGSR over Light_NGR were 0.92% and 0.4% on the Ciao and Epinions datasets, respectively. Compared with Light_NGR, the values of MAE achieved by Light_NGSR increased by 1.75% and 1.15% on the Ciao and Epinions datasets, respectively. This experimental results indicate that the integration of social network information is able to contribute to the recommendation quality of our proposed recommendation model. We argue that the underlying reason is that not only does Light_NGSR capture the high-order collaborative signals over the user–item interaction graph, but it also captures the high-order collaborative signal over the social network graph. By injecting high-order collaborative signals hidden in the social network graph, the representations of users in the social network are able to mutually reinforce one another. Hence, the semantics of the users’ representations are further enriched.

In addition, social network information had a more significant effect with Ciao than with Epinions. In other words, the improvements of Light_NGSR over Light_NGR on Ciao were larger than those on Epinions. The main reason is that the social network in Ciao is denser than that in Epinions. This finding indicates that a dense social network is beneficial to our proposed recommendation model.

### 5.7. The Impact of the Embedding Dimension *K*

**Figure 4:**
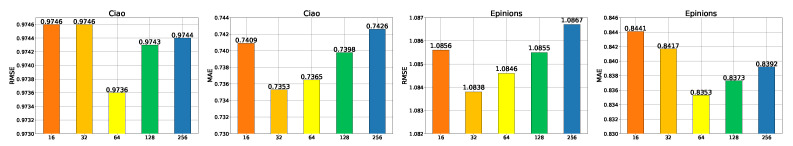
The performance comparison of the different embedding dimensions *K* of this model on the Ciao and Epinions datasets.

The embedding dimension *K* is an important factor that affects the performance of our proposed model. In this section, we varied the value of *K* among the values of [16, 32, 64, 128, 256] and investigated the impact of the embedding dimension on recommendation quality. The experimental results are shown in Figure 4.

As can be seen, the recommendation performance of our proposed method first improved and then degraded as we continued to increase the values of *K*. Light_NGSR achieved its best performance when *K* was around 32 or 64. Moreover, larger values of *K* did not ensure the improvement of recommendation performance. This observation conforms to the underlying assumption of matrix-factorization-based recommendation models, where only a few latent factors contribute to users’ preferences and items’ characteristics.

### 5.8. Comparison of the Training Efficiency

**Figure 5:**
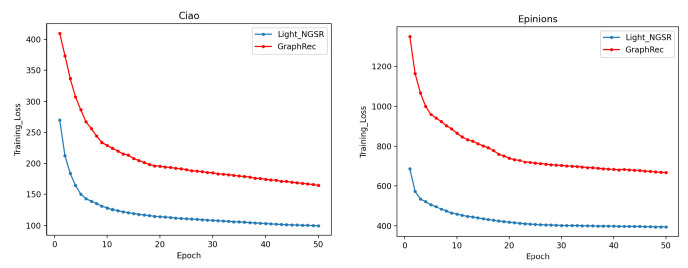
Training efficiency comparison.

In order to ease the model training, our proposed recommendation model adopts a lightweight GNN framework to aggregate neighborhood information. In this section, we describe a group of experiments that we conducted to compare the training efficiency of Light_NGSR with that of GraphRec. The parameter settings were the same as those in Section 5.4. The training curves of the training loss are plotted in Figure 5.

As shown in Figure 5, the training loss of Light_NGSR was consistently lower than that of GraphRec at each epoch. Moreover, on Ciao, our proposed recommendation model basically converged at the 15th epoch, while GraphRec basically converged at the 25th epoch. We also observed a similar trend on Eipions. Hence, in terms of training efficiency, our proposed method outperformed GraphRec. According to the combined results of the training efficiency comparison and the recommendation performance comparison, we argue that the lightweight GNN framework was able to ease the training process of GNN-based social recommendation models and alleviate overfitting.

## 6. Conclusions

Traditional social recommendation methods ignore high-order collaborative signals when formulating users’ and items’ representations. To tackle this issue, in this paper, we proposed a novel GNN-based social recommendation model that utilizes the GNN framework to capture high-order collaborative signals in the process of learning the latent representations of users and items. Specifically, we formulated the representations of users and items by stacking multiple embedding propagation layers to recursively aggregate the representations of multi-hop neighbors on both the user–item interaction graph and the social network graph. In this way, the final representations of users and items simultaneously encode the collaborative signal hidden in both the user–item interaction graph and the social network graph, thus significantly enriching the semantics of the representations of users and items. Moreover, we adopted a lightweight GNN framework to aggregate the neighborhood information, which eased the training process of the proposed GNN-based social recommendation model and alleviated the problem of overfitting. The experimental results on two real-world datasets show that our proposed method outperformed the state-of-the-art recommendation algorithms.

Our proposed recommendation method basically treats each trusted friend of a user equally when aggregating neighborhood information over the social network. In other words, we only considered the local structure of the social network and ignored the global structure when assigning the weight to each trusted user. In fact, each trusted friend of a user has a different authority, and authoritative users have a greater influence on their trustees. Hence, different trusted friends should be assigned different weights in the process of neighborhood information aggregation. In the future, we plan to utilize the PageRank algorithm to compute the rank of each user in the social network and integrate personalized weights derived from the ranks of users into our proposed recommendation model to further boost the recommendation performance.

## Figures and Tables

**Table 1:** Statistics of the datasets.

Dataset	Ciao	Epinions
Users	7375	40,163
Items	105,114	139,738
Ratings	284,086	664,824
Density (Ratings)	0.04%	0.01%
Social Connections	111,781	442,980

**Table 2:** Parameter settings of the methods used for comparison.

Algorithms	Parameter Settings
PMF	$\lambda_{U}$ = $\lambda_{V}$ = 0.001
SoRec	$\lambda_{U}$ = $\lambda_{V}$ = $\lambda_{Z}$ = 0.001, $\lambda_{C}$ = 1
RSTE	$\lambda_{U}$ = $\lambda_{V}$ = 0.001, α = 0.4
SocialMF	$\lambda_{U}$ = $\lambda_{V}$ = 0.001, $\lambda_{T}$ = 1
TrustMF	λ = 0.001, $\lambda_{T}$ = 1
GraphRec	λ = 0.001
Light_NGSR	λ = 0.001

**Table 3:** Performance comparison on Ciao.

Algorithms	RMSE	MAE
PMF	2.1294	1.6474
SoRec	1.0267	0.7985
RSTE	1.0304	0.8191
SocialMF	1.0143	0.7865
TrustMF	1.0599	0.7968
GraphRec	0.9859	0.7679
Light_NGSR	**0.9736**	**0.7365**

**Table 4:** Performance comparison on Epinions.

Algorithms	RMSE	MAE
PMF	1.9160	1.4424
SoRec	1.1192	0.8693
RSTE	1.1053	0.8844
SocialMF	1.0995	0.8656
TrustMF	1.1854	0.9340
GraphRec	1.0867	0.8488
Light_NGSR	**1.0846**	**0.8353**

## Data Availability

This research employed publicly available datasets for its experimental studies.
